# Glycosylation of a Capsule-Like Complex (CLC) by *Francisella novicida* Is Required for Virulence and Partial Protective Immunity in Mice

**DOI:** 10.3389/fmicb.2017.00935

**Published:** 2017-05-30

**Authors:** Kelly C. Freudenberger Catanzaro, Anna E. Champion, Nrusingh Mohapatra, Thomas Cecere, Thomas J. Inzana

**Affiliations:** ^1^Department of Biomedical Sciences and Pathobiology, Center for Molecular Medicine and Infectious Diseases, Virginia-Maryland College of Veterinary Medicine, Virginia Tech Blacksburg, VA, United States; ^2^Department of Biomedical Sciences, Virginia Tech Carilion School of Medicine Roanoke, VA, United States

**Keywords:** *Francisella novicida*, capsule-like complex, virulence in mice, tularemia, glycosylation, protective immunity

## Abstract

*Francisella tularensis* is a Gram-negative bacterium and the etiologic agent of tularemia. *F. tularensis* may appear encapsulated when examined by transmission electron microscopy (TEM), which is due to production of an extracellular capsule-like complex (CLC) when the bacterium is grown under specific environmental conditions. Deletion of two glycosylation genes in the live vaccine strain (LVS) results in loss of apparent CLC and attenuation of LVS in mice. In contrast, *F. novicida*, which is also highly virulent for mice, is reported to be non-encapsulated. However, the *F. novicida* genome contains a putative polysaccharide locus with homology to the CLC glycosylation locus in *F. tularensis*. Following daily subculture of *F. novicida* in Chamberlain's defined medium, an electron dense material surrounding *F. novicida*, similar to the *F. tularensis* CLC, was evident. Extraction with urea effectively removed the CLC, and compositional analysis indicated the extract contained galactose, glucose, mannose, and multiple proteins, similar to those found in the *F. tularensis* CLC. The same glycosylation genes deleted in LVS were targeted for deletion in *F. novicida* by allelic exchange using the same mutagenesis vector used for mutagenesis of LVS. In contrast, this mutation also resulted in the loss of five additional genes immediately upstream of the targeted mutation (all within the glycosylation locus), resulting in strain *F. novicida* Δ1212–1218. The subcultured mutant *F. novicida* Δ1212–1218 was CLC-deficient and the CLC contained significantly less carbohydrate than the subcultured parent strain. The mutant was severely attenuated in BALB/c mice inoculated intranasally, as determined by the lower number of *F. novicida* Δ1212–1218 recovered in tissues compared to the parent, and by clearance of the mutant by 10–14 days post-challenge. Mice immunized intranasally with *F. novicida* Δ1212–1218 were partially protected against challenge with the parent, produced significantly reduced levels of inflammatory cytokines, and their spleens contained only areas of lymphoid hyperplasia, whereas control mice challenged with the parent exhibited hypercytokinemia and splenic necrosis. Therefore, *F. novicida* is capable of producing a CLC similar to that of *F. tularensis*, and glycosylation of the CLC contributed to *F. novicida* virulence and immunoprotection.

## Introduction

The Gram-negative, intracellular bacterium *Francisella tularensis* causes the disease tularemia in numerous animal species and humans (Sjöstedt, 2005). Humans can acquire *F. tularensis* naturally through the bite of an arthropod vector such as a tick, the inhalation of aerosolized bacteria, the handling of an infected carcass, consumption of contaminated water or food, or laboratory exposure (Dennis et al., 2001; Metzger et al., 2007). Manifestations of tularemia in humans depend on the route of inoculation and the infectious strain. *F. tularensis* subspecies *tularensis* (Type A) is responsible for more severe disease, including pneumonic tularemia and potential mortality, whereas subspecies *holarctica* (Type B) causes less severe disease (Sjöstedt, 2007). Type A isolates are found exclusively in North America and as few as 10 bacterial cells can cause human disease (Saslaw et al., 1961; Sjöstedt, 2007). Both subspecies *tularensis* and *holarctica* are considered Tier I Select Agents by the Center for Disease Control and Prevention (CDC) due to their low infectious dose, high level of virulence, and ease of dispersal (Centers for Disease Control and Prevention and Department of Health and Human Services, 2012). The attenuated live vaccine strain (LVS) was derived from *F. tularensis* subspecies *holarctica* and is exempt from Select Agent regulations (Centers for Disease Control and Prevention and Department of Health and Human Services, 2012). Furthermore, the LVS genome contains multiple mutations and is no longer used as a vaccine candidate due to strain instability and potential virulence for immunocompromised individuals (Conlan and Oyston, 2007).

Several virulence factors of *F. tularensis*, including capsules, lipopolysaccharide (LPS), type IV pili, several secretion systems, outer membrane proteins, the *Francisella* pathogenicity island, and associated proteins, and others, have been extensively studied and reviewed (Jones et al., 2014; Rowe and Huntley, 2015). Unlike the LPS of enteric Gram-negative bacteria, the LPS of *F. tularensis* does not signal through, and is not an agonist of, toll-like receptor 4 (TLR-4) and has little endotoxic activity (Gunn and Ernst, 2007). Loss of the LPS O-antigen severely attenuates *F. tularensis* in the mouse model and antibodies to the LPS provide some protection against challenge with Type B strains, but not against Type A strains (Li et al., 2007; Raynaud et al., 2007; Modise et al., 2012). *F. tularensis* strains have also been reported to be encapsulated, based on identification of an electron dense material surrounding the cells by transmission electron microscopy (TEM; Sandström et al., 1988; Cherwonogrodzky et al., 1994; Sjöstedt, 2005; Bandara et al., 2011). Enhanced encapsulation by serial culture of the bacteria in Chamberlain's defined medium (CDM) increases the virulence of LVS for mice (Cherwonogrodzky et al., 1994). This electron dense capsule is visible by negative staining TEM around *F. tularensis* Type A and Type B strains, and is referred to as a large molecular size capsule-like complex (CLC; Bandara et al., 2011). The CLC appears to be a mixture of upregulated proteins, many of which are glycosylated, and are of large molecular size, and distinct from *F. tularensis* LPS or the O-antigen capsular polysaccharide, which is only visible around the cells when bound to labeled, specific antibodies (Apicella et al., 2010; Bandara et al., 2011; Champion et al., 2015, unpublished data). Daily passage of *F. tularensis* in CDM broth, followed by culture on CDM agar (CDMA) at about 30°C results in enhanced expression of the CLC. In contrast, no detectable CLC is produced in culture supernatant or around the cells when *F. tularensis* is grown in broth to mid- or late-log phase (Bandara et al., 2011). Deletion of two glycosyltransferase genes in a glycosylation locus distinct from the O-antigen locus in LVS or SchuS4 (Larsson et al., 2005) results in loss of the CLC on the cell surface and attenuation of LVS in mice. Immunization with this deletion mutant (LVSΔ1422–23) also protects mice against lethal challenge with virulent LVS (Bandara et al., 2011).

*F. tularensis* subspecies *novicida* (referred to here as *F. novicida*) retains a high genetic identity to the more virulent subspecies, is highly virulent for mice, but is only considered virulent for compromised humans (Keim et al., 2007). Unlike subspecies *tularensis* and *holarctica, F. novicida* is reported to be non-encapsulated (Sjöstedt, 2005; Elkins et al., 2007; Barker et al., 2009), and does not produce the O-antigen capsule described for LVS (Apicella et al., 2010). Nonetheless, we have identified a locus in *F. novicida* (FTN_1211-FTN_1221) with substantial homology to the locus involved in CLC glycosylation of *F. tularensis* (FT0789-FT0800). Therefore, due to the large degree of genetic identity between *F. novicida* and *F. tularensis* (~97%; Kingry and Petersen, 2014), the lack of mutations in wildtype *F. novicida* (as there are in LVS), and that *F. novicida* is as virulent as type A *F. tularensis* for the mouse (the most common model for studying tularemia), but relatively easy to modify genetically, clarifying the presence and role of the CLC in *F. novicida* will aid in clarifying its role as a virulence factor in type A *F. tularensis*. In this investigation we showed that when *F. novicida* was grown under conditions that enhance expression of the CLC in *F. tularensis* (Bandara et al., 2011), an electron dense material was also detected surrounding *F. novicida*. A *F. novicida* mutant was generated that lacked multiple genes within the glycosylation locus (FTN_1212–1218). This mutant was CLC-deficient, attenuated in a mouse model of tularemia, and the live mutant provided limited protection to mice against challenge with wild-type *F. novicida*.

## Materials and methods

### Bacterial strains and growth conditions

The bacterial strains and plasmids used in this study are listed in Table 1. *Francisella* strains were grown on Chamberlain's defined medium agar (CDMA; Chamberlain, 1965) or brain heart infusion agar (BD, Franklin Lakes, N.J.) containing 0.1% cysteine (BHI-C) at 37°C with 6% CO_2_. Broth cultures of *Francisella* strains were grown in Chamberlain's defined medium broth (CDMB; Chamberlain, 1965) or BHI-C broth with shaking (175–200 rpm) at 37°C, unless otherwise indicated. Type A strain TI0902 was grown in the CDC-certified BSL-3 laboratory at the Center for Molecular Medicine and Infectious Diseases (CMMID). For enhanced expression of any potential CLC, all *Francisella* strains were subcultured daily in CDMB for 10 days (identified by extension name _P10), and then grown on CDMA for 5 days at 30–32°C with ~6% CO_2_, as described (Bandara et al., 2011). *Escherichia coli* strains were grown at 37°C in Luria-Bertani (LB) broth or on LB agar (BD). Antibiotics included for growth of recombinant *F. novicida* and *E. coli* strains were 20 and 50 μg/ml of kanamycin, or 250 and 100 μg/ml of hygromycin, respectively.

**Table 1:** **Bacterial strains used in this study**.

**Strain/Plasmid**	**Description**	**Source**
*F. novicida*	U112 wild type strain	Dr. Karen Elkins
*F. novicida*_P10	*F. novicida* subcultured 10 times in CDMB	This work
*F. novicida* Δ1212–1218	*F. novicida* with a deletion spanning FTN_1212 through FTN_1218; insertion of kanamycin resistance cassette	This work
*F. novicida* Δ1212–1218_P10	*F. novicida* Δ1212–1218 subcultured 10 times in CDMB	This work
tnfn1_pw060323p05q162	*F. novicida* strain with a T20 transposon insertion in FTN_1212	Gallagher et al., 2007
tnfn1_pw060323p03q152	*F. novicida* strain with a T20 transposon insertion in FTN_1213	Gallagher et al., 2007
tnfn1_pw060328p06q149	*F. novicida* strain with a T20 transposon insertion in FTN_1214	Gallagher et al., 2007
tnfn1_pw060323p05q110	*F. novicida* strain with a T20 transposon insertion in FTN_1215	Gallagher et al., 2007
tnfn1_pw060420p04q184	*F. novicida* strain with a T20 transposon insertion in FTN_1216	Gallagher et al., 2007
tnfn1_pw060418p03q107	*F. novicida* strain with a T20 transposon insertion in FTN_1217	Gallagher et al., 2007
tnfn1_pw060323p07q127	*F. novicida* strain with a T20 transposon insertion in FTN_1218	Gallagher et al., 2007
Type A *F. tularensis* strain TI0902	Type A wildtype strain	Inzana et al., 2004
LVS	*F. tularensis* subspecies *holarctica* live vaccine strain	Dr. May Chu
LVS_P10	LVS subcultured 10 times in CDMB	Bandara et al., 2011
*E. coli* DH5α	Genotype: F^−^ Φ80*lac*ZΔM15 Δ(*lac*ZYA-*arg*F) U169 *rec*A1 *end*A1 *hsd*R17(${\text{r}}_{k}^{-}$, ${\text{m}}_{k}^{+}$) *pho*A*sup*E44 *thi*-1 *gyr*A96 *rel*A1 λ^−^	Invitrogen
*E. coli* Top10	Genotype**:** F- *mcrA* Δ(*mrr-hsd*RMS-*mcr*BC) Φ80*lac*ZΔM15 Δ *lac*X74 *rec*A1 *ara*D139 Δ(*araleu*)7697 *gal*U *gal*K *rps*L (StrR) *end*A1 *nup*G	Invitrogen
pSC-1423/1422K	*Francisella* suicide vector containing flanking regions to FTL_1423-22 with kanamycin resistance	Bandara et al., 2011
pMP822	Hygromycin resistant *E. coli*/*Francisella* shuttle vector with a *blab* promoter	LoVullo et al., 2009
pMP_FTN1212-1213	pMP822 with FTN1212-1213 inserted downstream of the *blab* promoter	This work

### Blast analysis of the *F. novicida* genome

The presence of a putative glycosylation locus distinct from the O-antigen locus in *F. novicida* was searched for using BLAST (Altschul et al., 1990) using the *F. tularensis* Type A SchuS4 glycosylation locus (FTT_0789 to FTT_0800) for comparison.

### Mutagenesis of *F. novicida*

Suicide plasmid pSC-1423/1422K, which was used for mutagenesis of LVS, was also used for mutagenesis of *F. novicida* by allelic exchange, targeting FTN_1212 and FTN_1213 (Bandara et al., 2011). Plasmid DNA was purified from *E. coli* using the QIAprep Spin Miniprep Kit (QIAGEN, Valencia, CA). The chemical transformation protocol used to introduce pSC-1423/1422K into *F. novicida* was based on a combination of previously described methods (Frank and Zahrt, 2007; Gallagher et al., 2008). An overnight culture of *F. novicida* grown on CDMA was suspended in 1 ml of phosphate buffered saline (PBS), pH 7.6. Approximately 100 ng of the plasmid DNA and 100 μl of the bacteria in PBS were added to 1 ml of transformation buffer (0.04% L-arginine, 0.04% L-aspartic acid, 0.02% L-histidine, 0.04% DL-methionine, 0.004% spermine phosphate, 1.58% sodium chloride, 0.294% CaCl2, and 0.6% Trizma base). This suspension was incubated at 37°C with slow agitation (100 rpm) for 1 h, 2 ml of CDMB was added, and incubation continued for 4–6 h with shaking at 200 rpm. Various concentrations of the culture were inoculated to selective BHI-C agar and incubated at 37°C in 6% CO_2_ for up to 5 days. Kanamycin-resistant colonies were screened for the correct insertion by PCR, and one mutant was confirmed to contain a deletion spanning at least FTN_1212 and FTN_1213 by a second round of PCR and by reverse transcriptase PCR (RT-PCR). To our surprise, subsequent analysis by PCR and RT-PCR indicated that in addition to FTN_1212–1213, the additional open reading frames (ORFs) FTN_1214–1218, which all reside within this putative glycosylation locus, were also absent. Therefore, the mutant was named *F. novicida* Δ1212–1218.

### PCR and DNA sequencing

A typical PCR reaction consisted of 1x PCR HIFI SuperMix (Invitrogen), 0.02 μg genomic DNA as template, and 0.4 μM of each oligonucleotide primer in 50 μl of reaction mixture. The PCR cycling parameters used were 94°C for 2 min followed by 35 cycles of 94°C for 30 s, 52°C for 30 s, and 68°C for 2 min, and an additional extension for 5 min at 68°C. For sequencing of the putative CLC glycosylation locus the forward primer was FTN_1219_F and the reverse primer was FTN_1210_R (Table S1). Amplicons from LVS, *F. novicida* U112, and the mutant strain were sequenced using FTN_1219_forward and FTN_1210_reverse primers at the Biocomplexity Institute at Virginia Tech using the ABI 3730 sequencer. Sequence files were analyzed using NCBI nucleotide BLAST online program (Altschul et al., 1990).

### Reverse transcriptase-PCR (RT-PCR)

RNA was isolated from *F. novicida* and *F. novicida* Δ1212–1218 using the RNeasy Mini Kit (Qiagen). cDNA was generated using the SuperScript III First-Strand (ThermoFisher) synthesis system (Invitrogen) following the manufacturer's instructions. The primers used to amplify cDNA of genes FTN_1211 to FTN_1221 for RT-PCR are listed in Table S1.

### Purification of the putative CLC

*F. novicida* was grown in CDMB to just past mid-log phase, subcultured consecutively in fresh medium 10 times daily, and then grown on CDMA for 5 days at 32°C with ~7% CO_2_ to obtain strain *F. novicida*_P10. The strain was then extracted with 0.5% phenol as previously described (Bandara et al., 2011) and by a modified method using urea (Champion et al., 2015). Briefly, the bacteria were scraped off 10 agar plates and suspended in ~100 ml of 1 M urea. This suspension was incubated at room temperature for 15 min and sedimented by centrifugation at 10,000 × g for 15 min. The supernatant was subjected to ultracentrifugation at 40,000 × g for 4 h to overnight, and the subsequent supernatant was dialyzed through a membrane with a pore size of 50,000 kDa in 10 mM HEPES and 0.1% sodium dodecyl sulfate (SDS) twice, followed by dialysis in 10 mM HEPES once. Sodium acetate (30 mM final) was added to the dialyzed liquid, followed by addition of 3–5 volumes of 95% ethanol, and the mixture incubated overnight at −20°C to precipitate any large molecular size material. The precipitate was sedimented by centrifugation at 10,000 × g for 30 min and resuspended in ~25 ml of buffer containing 50 mM Trizma base, 10 mM CaCl_2_, 10 mM MgCl_2_, and 0.05% sodium azide. Ten microliters of RiboShredder™ RNase Blend (Epicenter, Madison, WI) and 25 μg/ml of DNase were added, the solution incubated at 37°C overnight, and the mixture extensively dialyzed through a 50,000 kDa membrane in 4 L of distilled water (4–5 changes). Any large molecular size material in the retentate was precipitated with ethanol, as above, and lyophilized. Any putative CLC from other strains and mutants that were subcultured in CDMB was extracted in the same manner.

### TEM

*Francisella* strains were subcultured in CDMB and finally on CDMA, as described above, to enhance expression of CLC, or grown in BHI-C broth with shaking to minimize CLC production. TEM of the bacteria was carried out as previously described (Bandara et al., 2011). Briefly, the bacteria were gently suspended and fixed in 0.1 M sodium cacodylate buffer with 2.5% glutaraldehyde, and incubated with end-over-end rotation at room temperature for 2 h. The suspension was allowed to adhere to formvar-coated grids for 5 min and then stained with 0.5% uranyl acetate. The grids were briefly washed with distilled water, dried, and the bacteria viewed on a Jeol JEM-1400 electron microscope. Type A strain TI0902 was subcultured in CDMB as described above, fixed in 0.1 M sodium cacodylate buffer with 2.5% glutaraldehyde, and incubated with end-over-end rotation at room temperature for 2 h before being stored at 4°C for 5 days. A sample was inoculated onto CDMA and allowed to incubate for 5 days at 37°C to ensure complete loss of bacterial viability before the cells were processed as described above.

### Compositional analysis of the putative CLC

The carbohydrate composition of the *F. novicida*_P10 extract was determined by combined gas chromatography-mass spectrometry (GC/MS) at the University of Georgia Complex Carbohydrate Research Center, as previously described (Merkle and Poppe, 1994; Bandara et al., 2011).

Crude urea extracts were used to compare the relative amounts of protein and/or carbohydrate present in the putative CLC from *F. novicida*_P10, *F. novicida* Δ1212–1218_P10, and each transposon (Tn) mutant with single mutations in FTN_1212, FTN_1213, FTN_1214, FTN_1215, FTM_1216, FTN_1217, FTN_1218 that were subcultured on CDM 10 times to enhance surface expression of CLC. Colonies from individual plates were suspended in 4 ml of 1 M urea and incubated at room temperature for 15 min. The bacteria were sedimented by centrifugation at 10,000 × g for 15 min, and the supernatant was carefully removed for further analysis. Pelleted cells were used to determine the wet weight of bacteria for each crude extract. The amount of protein and carbohydrate/g of bacterial wet weight of the 1 M urea extract was determined using the Pierce™ BCA Protein Assay Kit (Thermo Scientific, Waltham, MA), and anthrone assay (Scott and Melvin, 1953), respectively.

### Electrophoretic profile of the putative CLC

Electrophoretic profiles were resolved on NuPAGE® Novex® 4–12% Bis-Tris Protein Gels (Life Technologies) by electrophoresis at a constant voltage of 200 v for ~40 min. Gels were stained with the Pierce Silver Stain Kit (Thermo Scientific), or 0.25% Stains-All (Sigma-Aldrich, St. Louis, MO; Bandara et al., 2011), or both.

### Virulence and protective efficacy of *F. novicida* Δ1212–1218 in mice

Female BALB/c mice 6–8 weeks old (Charles River Laboratories, Wilmington, MA) were housed in an AALAC-accredited ABSL-2 facility. Groups of 7–8 mice were used to assess the virulence of *F. novicida* Δ1212–1218 compared to the parent strain. Mice were anesthetized with 3–4% isofluorane and inoculated intranasally (IN) with 50, 100, 1,000, or 10,000 CFU of *F. novicida* Δ1212–1218/mouse, or 1,000 CFU of *F. novicida* (>100 X the LD_50_)/mouse (Kieffer et al., 2003; Lauriano et al., 2004; Cong et al., 2009), determined spectrophotometrically, in 20 μl of PBS. All inoculation doses were confirmed by viable plate count on BHI-C agar. Mice were monitored daily, scored for health status, and weighed daily after inoculations. Moribund mice were euthanized with excess CO_2_ and lungs, liver, and spleen were collected. Some mice were euthanized at 1, 3, 6, 10, and 14 days post-challenge. The number of bacteria in the liver, lungs, and spleen were determined by viable plate count of weighed, homogenized tissues.

Groups of four mice each were immunized IN with various doses of *F. novicida* Δ1212–1218 (50, 100, 1,000, or 10,000 CFU/mouse) in 20 μl of PBS or PBS alone. Six weeks after immunization the mice were challenged IN with 1,000 CFU of *F. novicida*. All mice were monitored for 14 days and then euthanized. Any animals that became moribund prior to day 14 post-challenge were euthanized immediately. Tissues were harvested from all mice and cultured to determine bacterial numbers from each inoculation group.

### Histopathology of spleen samples

Spleen samples were sent to the histopathology laboratory at the Virginia-Maryland College of Veterinary Medicine (VMCVM) for preparation and staining. Briefly, sections of spleen were fixed in 10% neutral buffered formalin, processed, and embedded in paraffin. Sections were stained with hematoxylin and eosin (H&E), read with an Olympus BX43 microscope, and photomicrographs were taken with an Olympus DP73 digital camera and cellSens software.

### Cytokine analyses

Spleen tissues from the challenged mice were lysed using the Bio-Plex Cell Lysis Kit according to the manufacturer's instructions (Bio-Rad). The Pierce™ BCA Protein Assay Kit (Thermo Scientific) was used to determine the total protein level of each lysate for standardization of samples. Lysates were analyzed in triplicate using the Bio-Plex Pro™ Mouse Cytokine Th1/Th2 Assay (Bio-Rad, Hercules, CA) following the manufacturer's instructions.

### Statistical analyses

Student's *T*-test was used to evaluate significant differences in the carbohydrate and protein composition of the putative CLC from *F. novicida*_P10 and *F. novicida* Δ1212–1218_P10. The carbohydrate content of subcultured *F. novicida* transposon mutants corresponding to each gene affected in the *F. novicida* Δ1212–1218 strain was compared to the subcultured parent strain using One-Way ANOVA. The Mantel-Cox log-rank test was used to compare the survival curves of the control and immunized mice following challenge. Multiple *T*-tests using the Holm-Sidak method for correcting multiple comparisons was used to assess differences in bacterial load in mice inoculated with *F. novicida* or *F. novicida* Δ1212–1218. One-way ANOVA was also used to evaluate significance in bacterial loads and cytokine levels of immunized mice compared to control mice. Tukey's *post-hoc* test was used after the completion of the one-way ANOVA to identify specific differences between the bacterial loads and cytokine levels of inoculation groups. Statistical analyses were determined using GraphPad Prism 6 (GraphPad Software Inc., La Jolla, CA).

## Results

### Extraction of putative CLC from *F. novicida*

**Figure 1:**
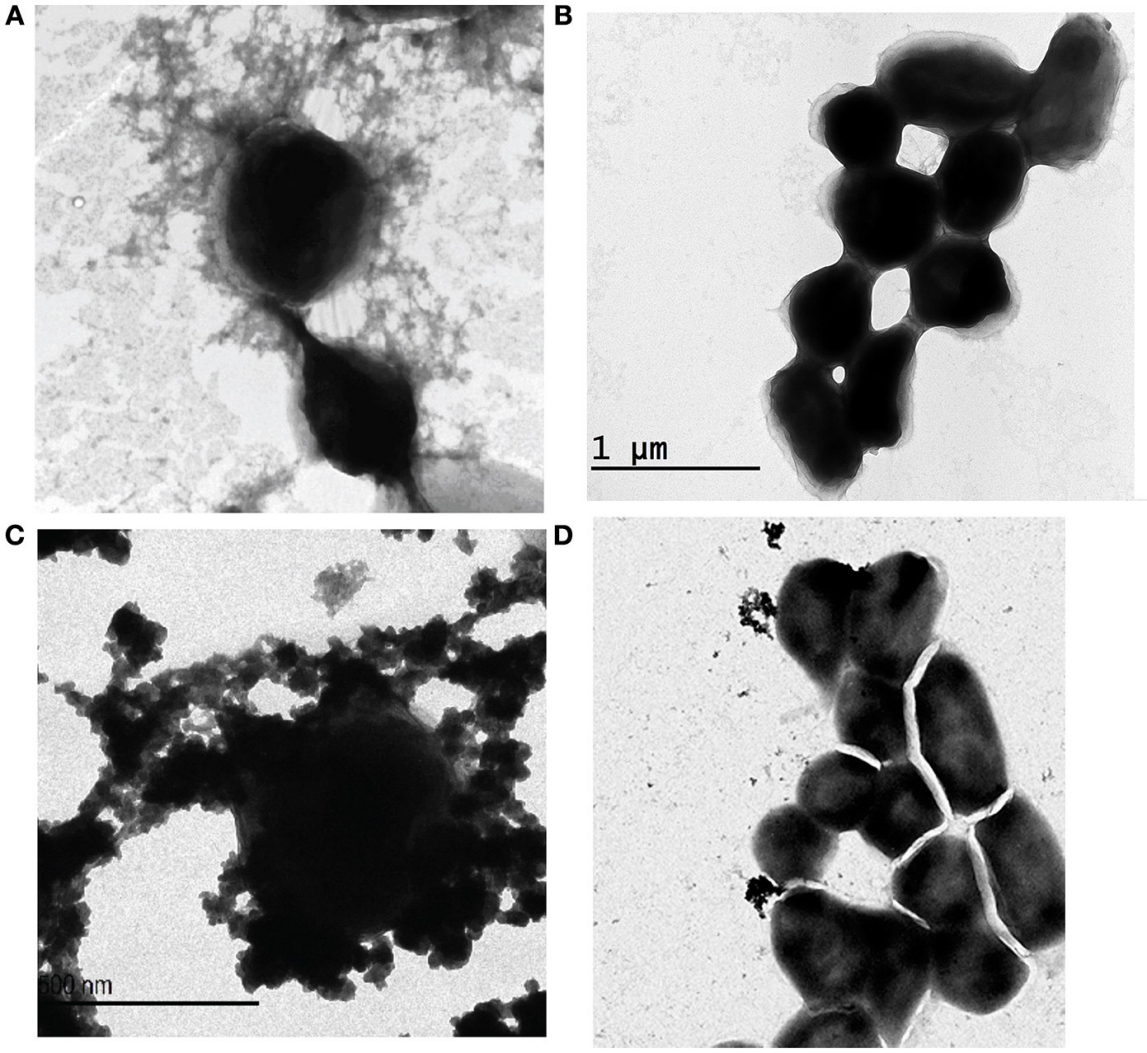
**Electron micrographs of ***Francisella*** subspecies and glycosyltransferase mutants**. Bacterial cells were negatively stained with uranyl acetate and examined by TEM. *Francisella* strains were subcultured in CDMB **(A,C,D)** for 10 consecutive days and then grown for 5 days at 32°C on CDMA for 5 days to enhance CLC expression. *F. novicida* grown in BHI-C broth with shaking to mid-log phase did not express this electron dense material **(B)**. *F. novicida* cells grown to enhance CLC expression **(C)** were surrounded by a similar electron dense material as that produced by *F. tularensis* Type A grown to enhance CLC expression **(A)**. The glycosyltransferase mutant *F. novicida* Δ1212–1218_P10 **(D)** produced little of the electron dense material, but what material was present was not closely associated with the cell surface. Micrographs shown are representative of most fields. Scale bars represent 500 nm.

Following serial passage of *F. novicida* strain U112 in CDMB and growth on CDMA for 5 days at 32°C, aggregates of an electron dense material were identified around the bacteria following negative staining and TEM (Figure 1C). The electron dense material surrounding *F. novicida*_P10 was similar to the aggregated material observed surrounding *F. tularensis* Type A strain TI0902_P10 (Figure 1A) and *F. tularensis* Type B strain LVS_P10 (Bandara et al., 2011). As reported for LVS (Bandara et al., 2011), *F. novicida* cells that were grown to mid-log phase in BHI-C broth at 37°C did not produce a visible electron dense material (Figure 1B).

**Figure 2:**
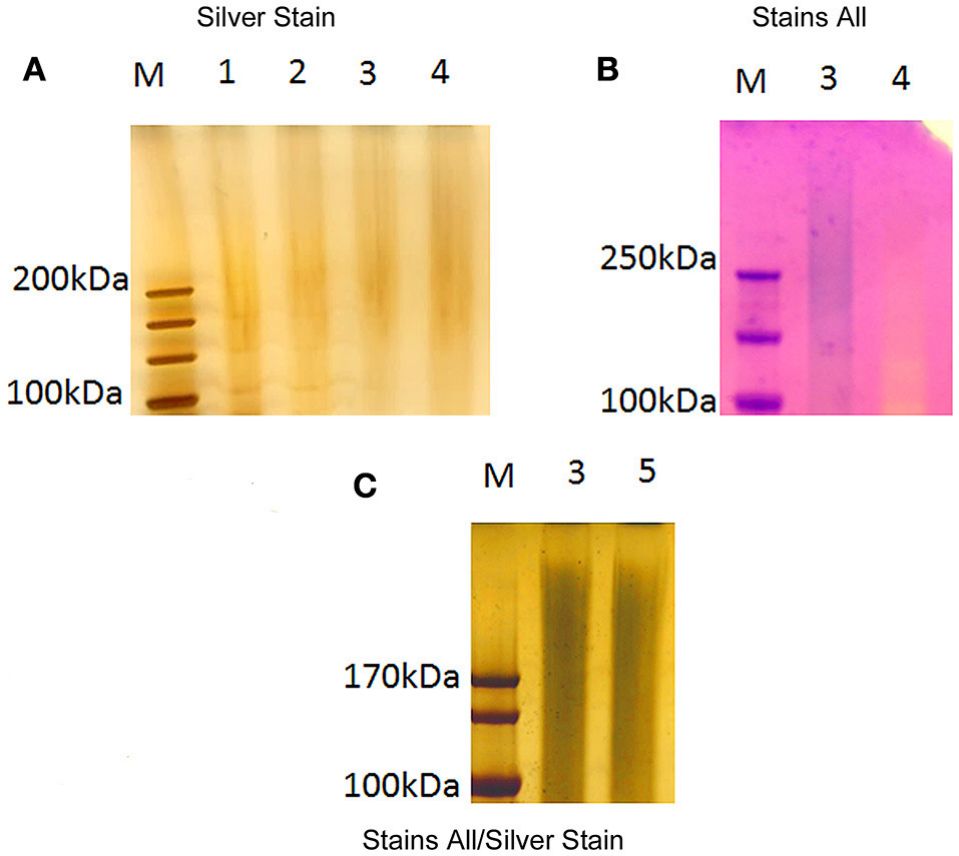
**Electrophoretic profile of large molecular size ***F. novicida*** extracts**. Extracts from *F. novicida* were separated by electrophoresis through a 4–12% SDS-PAGE gel. The gels were subsequently stained by silver stain **(A)**, Stains-All **(B)**, and Stains-All/silver **(C)**. LVS CLC was used for comparison. Analysis of the profiles focused on material >100 kDa. Lanes: M, molecular size standards; 1, LVS crude CLC in 1 M urea; 2, *F. novicida* crude extract in 1 M urea; 3 *F. novicida* soluble fraction extract (urea extracted); 4, *F. novicida* insoluble fraction extract (urea extracted); 5, *F. novicida* soluble fraction extract (phenol extracted).

The putative CLC from *F. novicida*_P10 was extracted as described in methods using 1 M urea in place of 0.5% phenol (Bandara et al., 2011). The use of urea improved solubility and diminished CLC aggregation for type A and B strains (Champion et al., 2015), which was problematic with the CLC extracted with phenol (Bandara et al., 2011). The electrophoretic profiles of *F. novicida*_P10 soluble fraction extracts using urea vs. phenol were similar (Figure 2C) and were similar to the profile of the CLC from LVS_P10, although the extracts stained poorly with only silver stain (Figure 2A). Of particular note were the similarities of the large molecular size band from the *F. novicida*_P10 extract (Figure 2A, lanes 2 and 3) and the *F. tularensis* LVS_P10 CLC (Figure 2A, lane 1). As previously shown with the CLC from LVS and type A strains (Bandara et al., 2011; Champion et al., 2015), a wide variety of proteins were isolated from both the crude and the enzyme-digested *F. novicida*_P10 extracts. The putative CLC proteins could be further divided into soluble and insoluble portions based on their solubility in water, but the large molecular size smear (~250 kDa) was present in only the soluble portion of the *F. novicida*_P10 extract and was highly visible using the cationic dye Stains-all (Figure 2B). Selective staining with Stains-All further supported that the material at ~250 kDa contained carbohydrate (stained blue), which is likely why the material stained poorly with only silver stain and appeared as a smear in the gels. GC-MS indicated that the carbohydrate portion of the putative CLC was composed of glucose and galactose in equal amounts, and less of mannose, which is identical to the glycoses identified in the LVS CLC (Bandara et al., 2011).

### Mutagenesis of the putative CLC glycosylation locus and reduction of the carbohydrate content in CLC extracts

**Figure 3:**
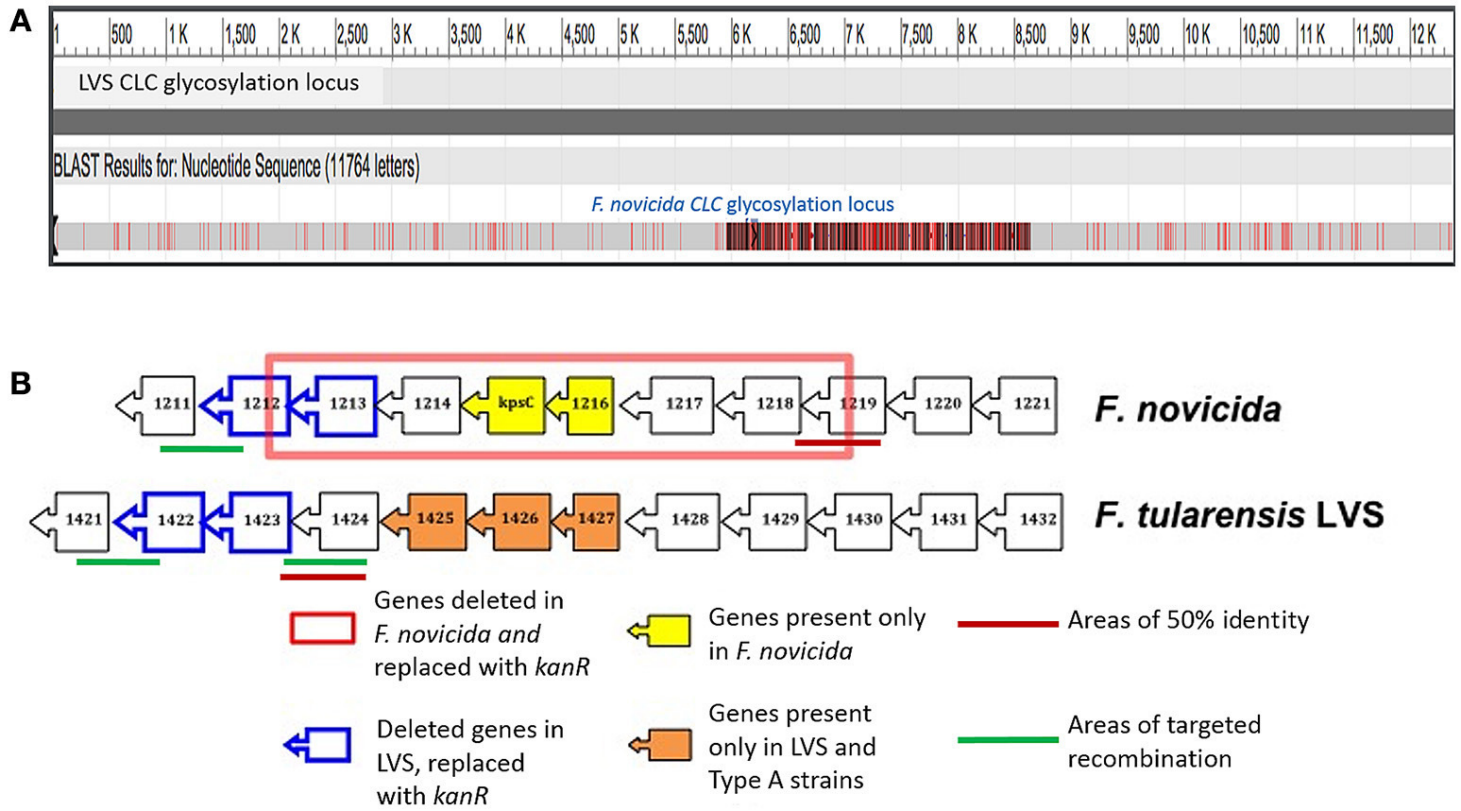
**Comparison of the CLC glycosylation loci of LVS and ***F. novicida***. (A)** Needleman-Wunsch alignment of the glycosylation loci of LVS with *F. novicida* showing the regions of dissimilarity in red. **(B)** Cartoon of the LVS and *F. novicida* CLC glycosylation loci showing the region deleted in *F. novicida* Δ1212–1218 (red box), genes unique to *F. novicida* and LVS (yellow and orange boxes, respectively), areas targeted for recombination using the same recombination vector used for LVS (green lines), areas of 51% identity where recombination actually occurred in *F. novicida* Δ1212–1218, and genes replaced with *kanR* gene in LVS and homologous genes targeted for deletion in *F. novicida* (blue boxes).

A DNA region in the genome of *F. novicida* U112 with homology to the LVS CLC glycosylation locus (Bandara et al., 2011) was identified by BLAST analysis (Table 2). The glycosylation locus in *F. tularensis* is comprised of 12 open reading frames (ORF), whereas the *F. novicida* locus contained 11 ORFs (Figure 3B). The *F. novicida* locus did not contain homologs to FTL_1425, FTL_1426, and FTL_1427, but did contain two alternate genes (FTN_1215 [*kpsC*] and FTN_1216) that are not found in *F. tularensis*. *F. novicida* did contain homologs to two genes required for CLC glycosylation in LVS (Bandara et al., 2011) and glycosylation of DsbA in type A *F. tularensis* (Thomas et al., 2011). These two genes (FTN_1212 and FTN_1213) were targeted for deletion in *F. novicida* to determine if they were also responsible for CLC glycosylation in *F. novicida*. Two genes were mutated because we previously showed that deletion of one gene (of two separate genes) in this locus failed to adequately delete glycosylation (Bandara et al., 2011).

**Table 2:** **CLC glycosylation locus in ***F. tularensis*** subspecies**.

***F. tularensis* subsp. *novicida* ORF**	**Size (bp)**	**Protein product^a^**	***F. tularensis* subsp. *holarctica* LVS ORF**	***F. tularensis* subsp. *tularensis* schuS4 ORF**	**% Sequence Identity to type A**
FTN_1221	669	D-ribulose-phosphate 3-epimerase	FTL_1432	FTT_0789	98
FTN_1220	1395	Glycosyltransferase	FTL_1431	FTT_0790	97
FTN_1219	1020	UDP-glucose 4-epimerase	FTL_1430	FTT_0791	97
FTN_1218	1230	Glycosyltransferase	FTL_1429	FTT_0792	97
FTN_1217	1689	ATP-binding membrane transporter	FTL_1428	FTT_0793	98
Not present	1287	Hypothetical protein	FTL_1427	FTT_0794	NA*^b^*
Not present	684	Hypothetical protein	FTL_1426	FTT_0795	NA
Not present	762	Hypothetical protein	FTL_1425	FTT_0796	NA
FTN_1216	744	Hypothetical protein with methyltransferase domain	Not present	Not present	NA
FTN_1215	1161	Capsule polysaccharide export protein	Not present	Not present	NA
FTN_1214	960	Galactosyltransferase	FTL_1424	FTT_0797	98
FTN_1213	1008	Galactosyltransferase	FTL_1423	FTT_0798	97
FTN_1212	1014	Mannosyltransferase	FTL_1422	FTT_0799	96
FTN_1211	663	Haloacid dehalogenase	FTL_1421	FTT_0800	98

a * F. novicida homologs to the F. tularensis Type A and LVS CLC glycosylation locus. Two genes are present in F. novicida that are not found in the F. tularensis locus. There are three genes in the F. tularensis CLC glycosylation locus that are not present in the F. novicida locus: FTT0794, FTT0795, and FTT0796; all three genes encode for hypothetical proteins*.

b *NA, Not applicable*.

**Figure 4:**
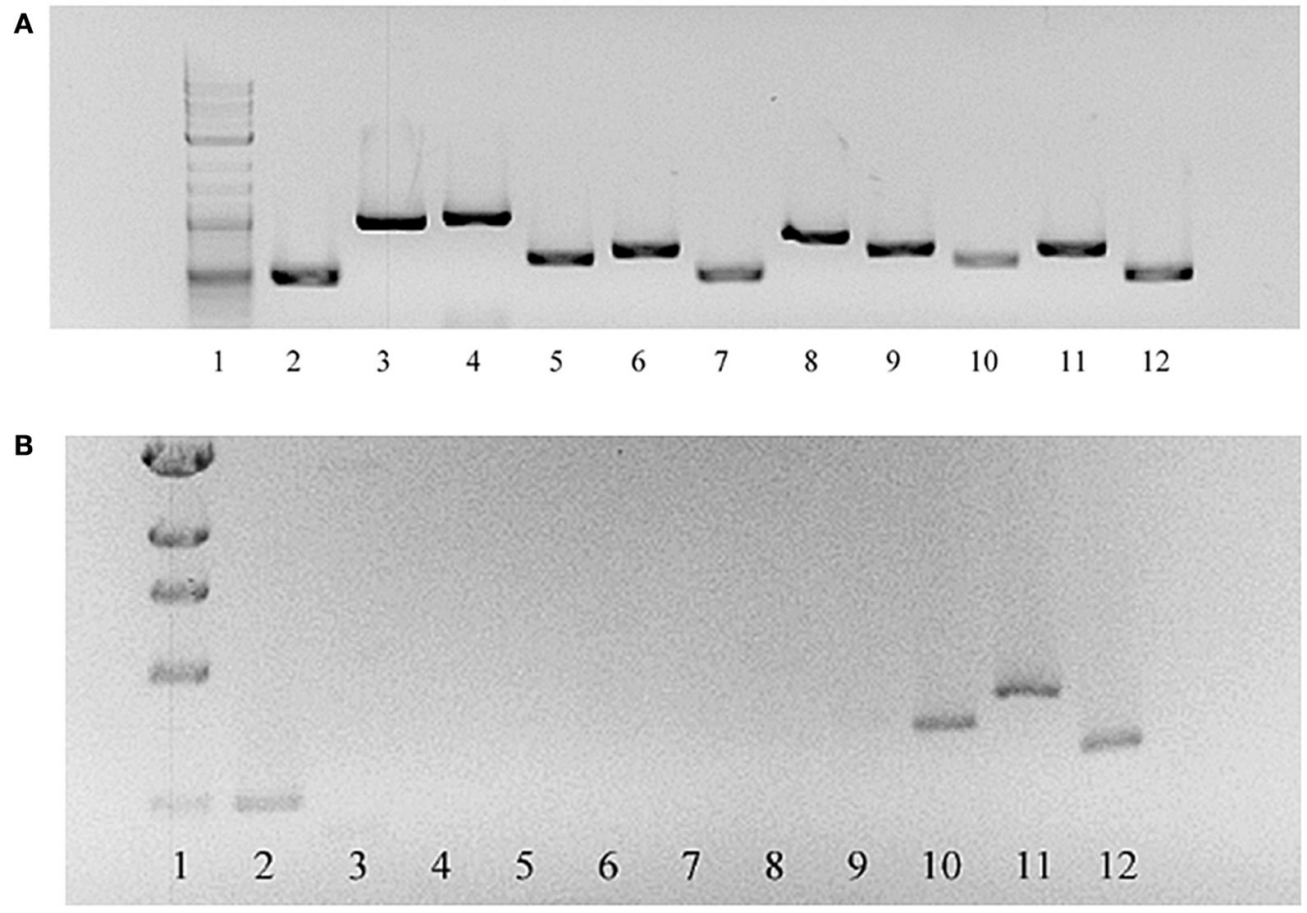
**RT-PCR of genes in the glycosylation locus of ***F. novicida*** Δ1212–1218 compared to the ***F. novicida*** parent**. RNA was isolated from both *F. novicida* and *F. novicida* Δ1212–1218 and converted to cDNA. Primers to each gene in the glycosylation locus were used to amplify gene products. Products were successfully amplified for the entire glycosylation locus in *F. novicida* **(A)**. Gene products were not amplified from FTN_1212 through FTN_1218 in *F. novicida* Δ1212–1218 **(B)**. Gene products from FTN_1219 through FTN_1221, also within the glycosylation locus, in *F. novicida* Δ1212–1218 were amplified. Lanes: 1, 1-kb ladder; 2–12, transcription products from genes FTN_1211–1221, respectively.

**Figure 5:**
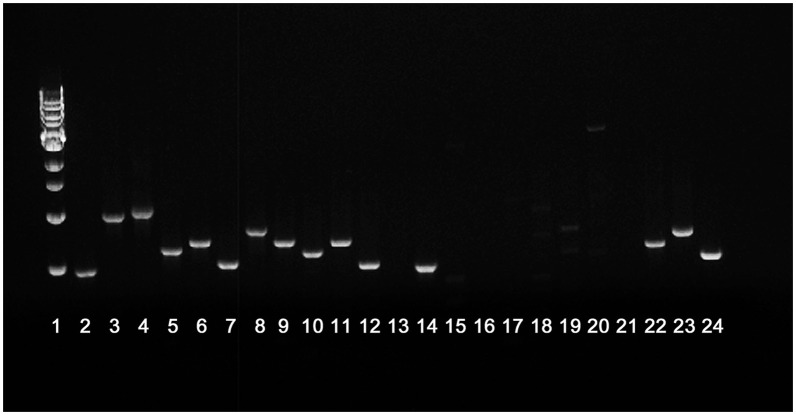
**PCR of each open reading frame within the putative CLC glycosylation locus**. Lanes 2–12 represent PCR amplification products from parent strain *F. novicida* U112. Lanes 14–24 represent PCR amplification products from mutant *F. novicida* ΔFTN_1212–1218. Lanes: 1, molecular size standards; 2 and 14, FTN_1211; 3 and 15, FTN_1212; 4 and 16, FTN_1213; 5 and 17, FTN_1214; 6 and 18, FTN_ 1215; 7 and 19, FTN_1216; 8 and 20, FTN_1217; 9 and 21, FTN_1218; 10 and 22, FTN_1219; 11 and 23, FTN_1220; 12 and 24, FTN_1221; 13, blank.

FTN_1212 and FTN_1213 were successfully deleted from *F. novicida* using allelic exchange with suicide plasmid pSC-1422/23K, as determined by amplification of the kanamycin resistance cassette and failure to amplify the targeted region by PCR or RT-PCR. However, RT-PCR of FTN_1212 and FTN_1213 and the remaining genes in this locus indicated that ORFs FTN_1212 through FTN_1218 were not expressed, but cDNA was amplified from the first (upstream) three genes in the locus: FTN_1219 to FTN_1221 and the gene downstream of the targeted mutation, FTN_1211, indicating the mutation was not polar and was restricted to the glycosylation locus (Figure 4B). Each of these genes in the parent were expressed normally (Figure 4A). Furthermore, PCR of each of the individual genes within the locus amplified FTN_1211 and FTN_1219–1221, but failed to amplify DNA from FTN_1212–1218, indicating that the allelic exchange mutation affected seven genes rather than only the two target genes (Figure 5). This mutant was named *F. novicida* Δ1212–1218. DNA sequencing of the entire locus showed that 100-bp of FTN_1219, all of FTN_1218–1213, and 210-bp of FTN_1212 were deleted and replaced with the kanamycin resistance gene (Figure 3B), confirming that FTN_1212–1218 were not functional (data not shown). Needleman–Wunsch global alignment of the glycosylation locus of LVS compared to *F. novicida* indicated there was 96–99% identity in 9 out of 12 genes present in the two loci (Figure 3A). Three LVS genes (FTL_1425–27) were missing in *F. novicida*, while two additional genes (*kpsC* and FTN_1216) were present exclusively in the locus of *F. novicida* (Figure 3A). DNA sequence analysis of the entire *F. novicida* glycosylation locus identified 1,019 nucleotides spanning FTN_1218 and FTN_1219 that shared 51% identity with the targeted region in FTL_1424 that flanks the LVS mutagenesis vector (Figure 3B, red line) where a recombination event occurred (not shown).

**Figure 6:**
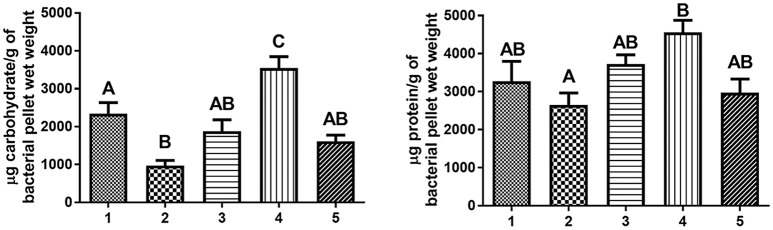
**Carbohydrate and protein content of urea extracts from ***F. novicida*** and LVS strains**. All strains were subcultured in CDMB and grown on CDMA at 32°C to enhance putative CLC production. The surface material was extracted using 1 M urea, and the crude CLC extracts were analyzed for carbohydrate content by the anthrone assay and for protein content by the BCA assay. The carbohydrate content of the extract from *F. novicida* Δ1212–1218_P10 contained significantly less carbohydrate than that of *F. novicida*_P10 (*p* = 0.02). The protein content between subcultured strains of *F. novicida* or between strains of LVS was not statistically different. Lanes: 1, *F. novicida*_P10; 2, *F. novicida* Δ1212–1218[1212–1213+]; 4, LVS_P10; 5, LVSΔ1422-1423_P10. Different letters above the bars indicate significant differences between the means when one-way ANOVA and Tukey's *post-hoc* were performed (*p* < 0.05). Identical uppercase letters indicate that there is no significant difference between the means. The value of a bar marked with “A” is significantly different from the values of bars marked with “B” or “C.” The values of bars marked “AB” are not statistically different from the values of bars marked with “A” or with “B,” but are statistically different from a bar marked with “C.”

*F. novicida* Δ1212–1218 and each of the 7 *F. novicida* transposon (TN) mutants with a single TN-interrupted gene in FTN_1212–1218 were subcultured ten times in CDMB to enhance any CLC material they were capable of making. The majority of *F. novicida* Δ1212–1218_P10 cells lacked any surface material around them, but some small amounts of putative CLC was observed by TEM surrounding *F. novicida* Δ1212–1218_P10. In contrast, the majority of the subcultured, *F. novicida* Δ1212–1218_P10 cells lacked any surface material around them. Furthermore, what surface material was present was not closely associated to the cells (Figure 1D), as it was on the surface of *F. novicida*_P10 (Figure 1C). The amount of protein in the extract from *F. novicida* Δ1212–1218_P10 was not significantly different from that found on *F. novicida*_P10, but the carbohydrate content of the putative CLC extract from *F. novicida* Δ1212–1218_P10 was significantly lower (*p* = 0.02; Figure 6), even though the LPS was not affected. However, the amount of carbohydrate present in the CLC from each of the subcultured isogenic TN mutants was not significantly different (*p* > 0.05) from that of the subcultured parent strain (Table S2). Therefore, mutagenesis of a single gene within this glycosylation locus was inadequate to significantly affect CLC glycosylation, as previously reported (Bandara et al., 2011).

### Attenuation of *F. novicida* Δ1212–1218 in mice

**Figure 7:**
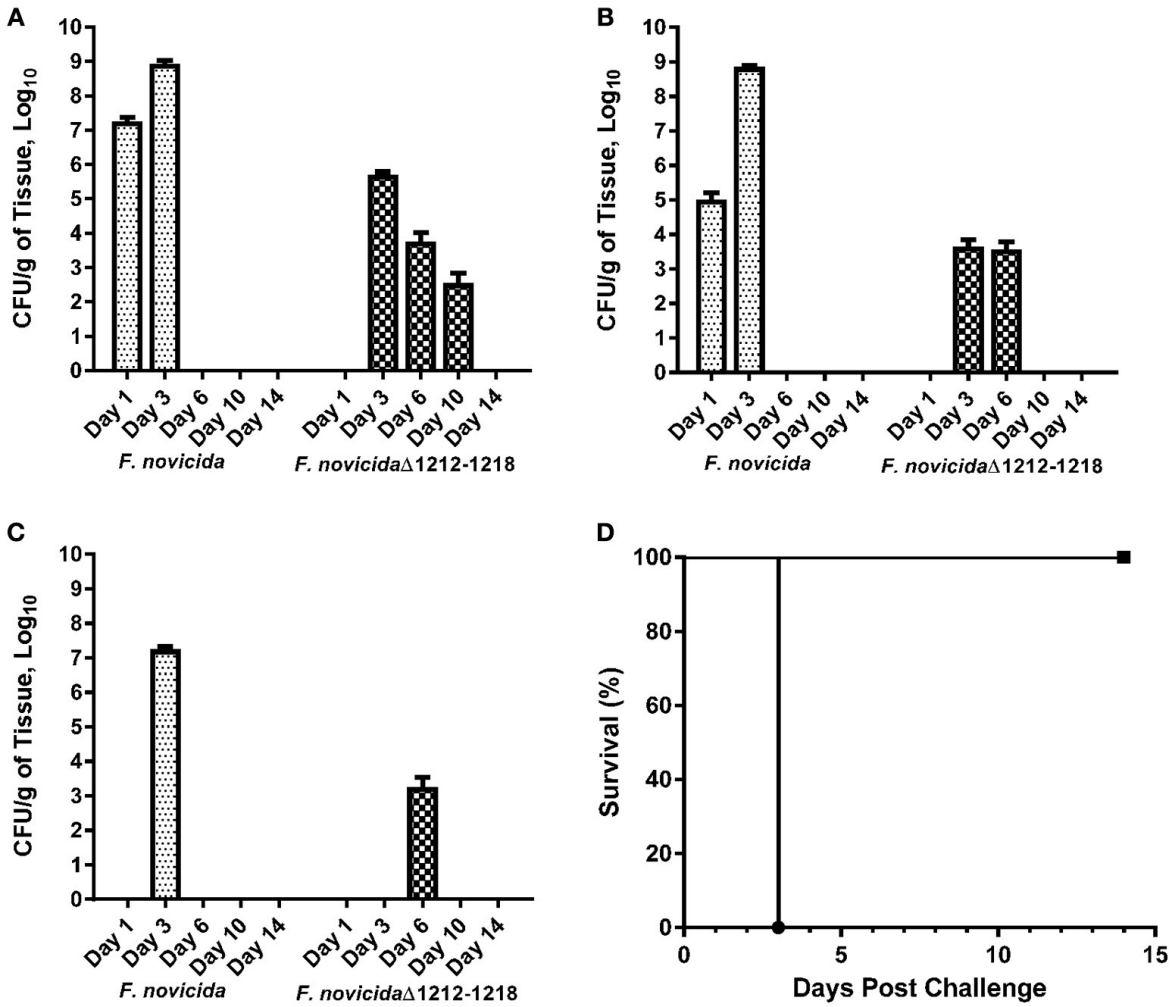
**Attenuation of ***F. novicida*** Δ1212–1218 in mice**. Mice were inoculated with 1000 CFU of wild-type *F. novicida* and *F. novicida* Δ1212–1218 IN, followed by euthanasia on days 1, 3, 6, 10, and 14. The bacterial burden in the lungs **(A)**, spleen **(B)**, and liver **(C)** were determined by viable plate counts. The bars represent the mean bacterial burden ± the SEM for *F. novicida* (dotted bar) and *F. novicida* Δ1212–1218 (checkered bar). All mice inoculated with *F. novicida* (•) needed to be euthanized by day 3. All mice inoculated with *F. novicida* Δ1212–1218 cleared the bacterial infection by day 10–14 **(D)**. *F. novicida* Δ1212–1218 (■) was attenuated in mice IN at a dose of at least 10,000 CFU and infection caused minimal to no signs of clinical illness.

Female BALB/c mice were inoculated IN with *F. novicida* or *F. novicida* Δ1212–1218 to determine if the lack of glycosylation affected virulence. All mice challenged with up to 10,000 CFU of *F. novicida* Δ1212–1218 IN survived the study duration of 14 days with minimal to no clinical signs (Figure 7D). Some groups of mice were inoculated with 1,000 CFU of *F. novicida* or *F. novicida* Δ1212–1218 IN and humanely euthanized at days 1, 3, 6, 10, and 14. The bacterial burden in the lungs (Figure 7A), liver (Figure 7B), and spleen (Figure 7C) increased significantly from days 1 to 3 in mice infected with *F. novicida* compared to mice challenged with *F. novicida* Δ1212–1218 (*p* = 0.0021, 0.0005, and <0.0001, respectively). Mice infected with *F. novicida* did not survive beyond 3 days post-challenge. Bacterial burdens on day 1 in the lungs, liver, and spleen of mice infected with *F. novicida* Δ1212–1218 were below the level of detection. Bacterial burdens in the lungs (Figure 7A) and spleen (Figure 7B) were detected on day 3, but were significantly lower (*p* = 0.0018 and > 0.001 respectively) than bacterial numbers detected on day 3 in mice infected with *F. novicida*. *F. novicida* Δ1212–1218 was detected in the liver on day 6, but not at other time points (Figure 7C). *F. novicida* Δ1212–1218 was undetectable in any organs of the mice by day 14 post-challenge. Therefore, *F. novicida* Δ1212–1218 was unable to multiply and disseminate in mice as efficiently as *F. novicida* and was significantly attenuated following challenge by the IN route of infection compared to *F. novicida* (*p* = 0.0183). Mice inoculated with 10,000 CFU of *F. novicida* Δ1212–1218 also had significantly less weight loss than mice inoculated with 1,000 CFU of *F. novicida* at 1, 3, and 4 days post-inoculation (*p* < 0.005; Figure S1).

### Protective efficacy of *F. novicida* Δ1212–1218 against challenge with the parent strain

**Figure 8:**
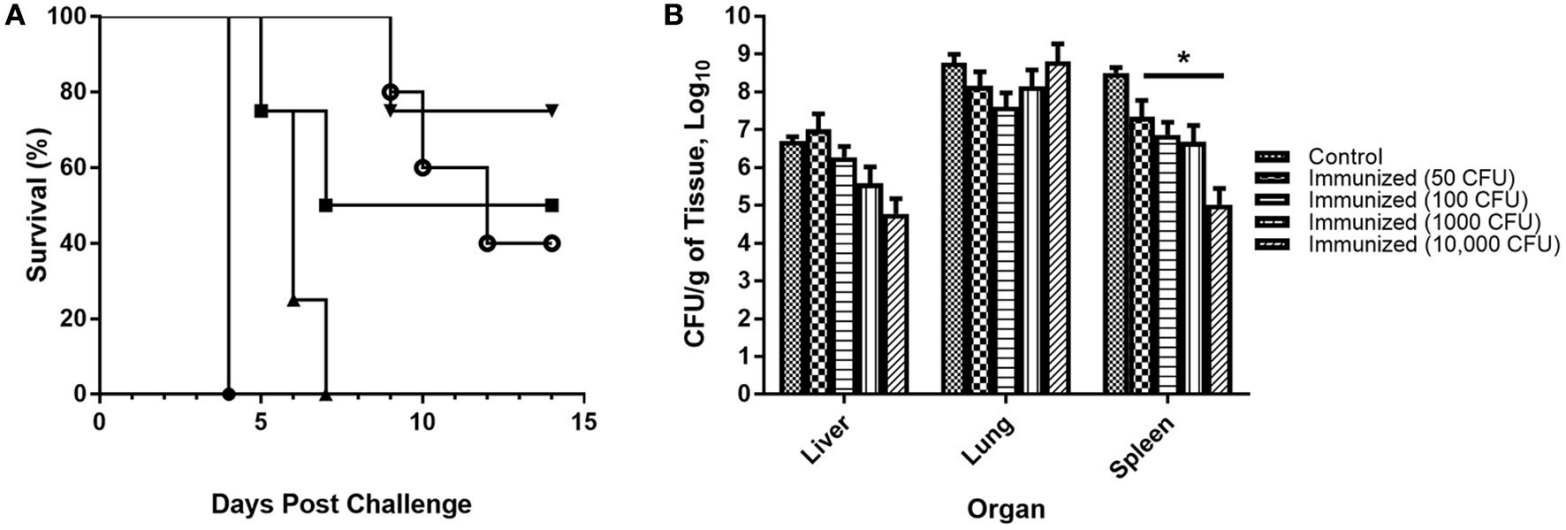
**Protection of mice immunized with ***F. novicida*** Δ1212–1218 against IN challenge with wildtype ***F. novicida***. (A)** BALB/c mice were immunized IN with varying doses of *F. novicida* Δ1212–1218 (50 CFU, ■; 100 CFU, ▴; 1000 CFU, ▾ and 10,000 CFU, °) or with PBS (•). Six weeks after immunization the mice were challenged IN with 1,000 CFU of *F. novicida* U112. Control mice immunized with PBS needed to be euthanized by day 4. Some of the mice in each group immunized with 50, 1,000, or 10,000 CFU of *F. novicida* Δ1212–1218 survived until day 14 post-challenge, which was statistically significant (*p* < 0.001) compared to the control group. **(B)** Bacterial numbers in the liver, lungs, and spleen were determined after euthanasia. All groups of immunized mice had significantly lower bacterial burdens in the spleen compared to control mice (^*^p < 0.0001).

BALB/c mice were immunized with PBS or variable doses of *F. novicida* Δ1212–1218 (50, 100, 1,000, and 10,000 CFU) IN. Six weeks after immunization the mice were challenged IN with 1,000 CFU of *F. novicida*, monitored for 2 weeks, and the survival of each group recorded (Figure 8A). All control mice became moribund between days 3 and 4 and were euthanized. All groups of immunized mice developed clinical symptoms following challenge with the parent strain and no group had 100% survival by day 14. Mice that developed more severe clinical symptoms, including hunched appearance and closed eyes, did not recover and were euthanized when the mice became moribund. Mice that experienced milder symptoms including slightly ruffled fur and some weight loss recovered by day 14, which included some mice in groups immunized with 50, 1,000, and 10,000 CFU of *F. novicida* Δ1212–1218; all surviving mice showed no clinical symptoms by day 14. All mice immunized with 100 CFU of *F. novicida* Δ1212–1218 needed to be euthanized by day 7 post-challenge.

Lungs, liver, and spleen were collected from each mouse post-mortem to determine the bacterial burden of *F. novicida* U112 in each organ (Figure 8B). No difference in bacterial burden was found in the liver and lungs of immunized mice compared to control mice, but immunized mice had significantly lower bacterial burdens in the spleen compared to control mice (*p* < 0.0001 for all immunized groups compared to control). Overall, immunized mice were partially protected from respiratory challenge with *F. novicida* U112, and the protection was dose dependent.

**Figure 9:**
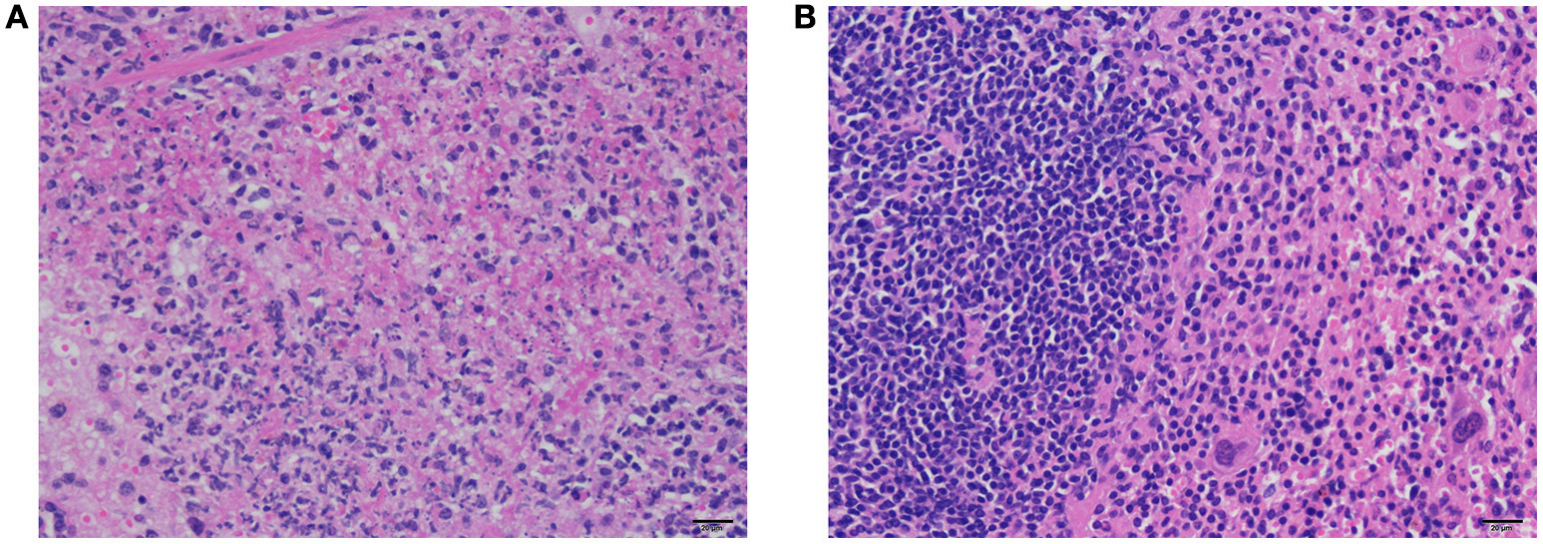
**Histopathology of spleens of immunized mice and control mice after challenge with ***F. novicida*****. There is necrosis and fibrin accumulation surrounded by neutrophils and cellular debris within the red pulp of spleens of control mice **(A)**. In contrast, there is moderate to marked lymphoid hyperplasia characterized by nodular aggregates of lymphocytes, but no evidence of necrosis or neutrophilic inflammation in the spleens of immunized mice **(B)**. H&E stain, bar = 20 μm.

Severe multifocal to coalescing fibrino-necrotizing splenitis was evident following histopathological examination of post-mortem spleens from control mice challenged with *F. novicida* (Figure 9A). However, there was lymphoid hyperplasia and extramedullary hematopoiesis in the spleens of mice immunized with *F. novicida* Δ1212–1218 and then challenged with *F. novicida* (Figure 9B). There was no evidence of necrosis or neutrophilic inflammation in the spleens of immunized mice, unlike the spleens of control mice.

**Figure 10:**
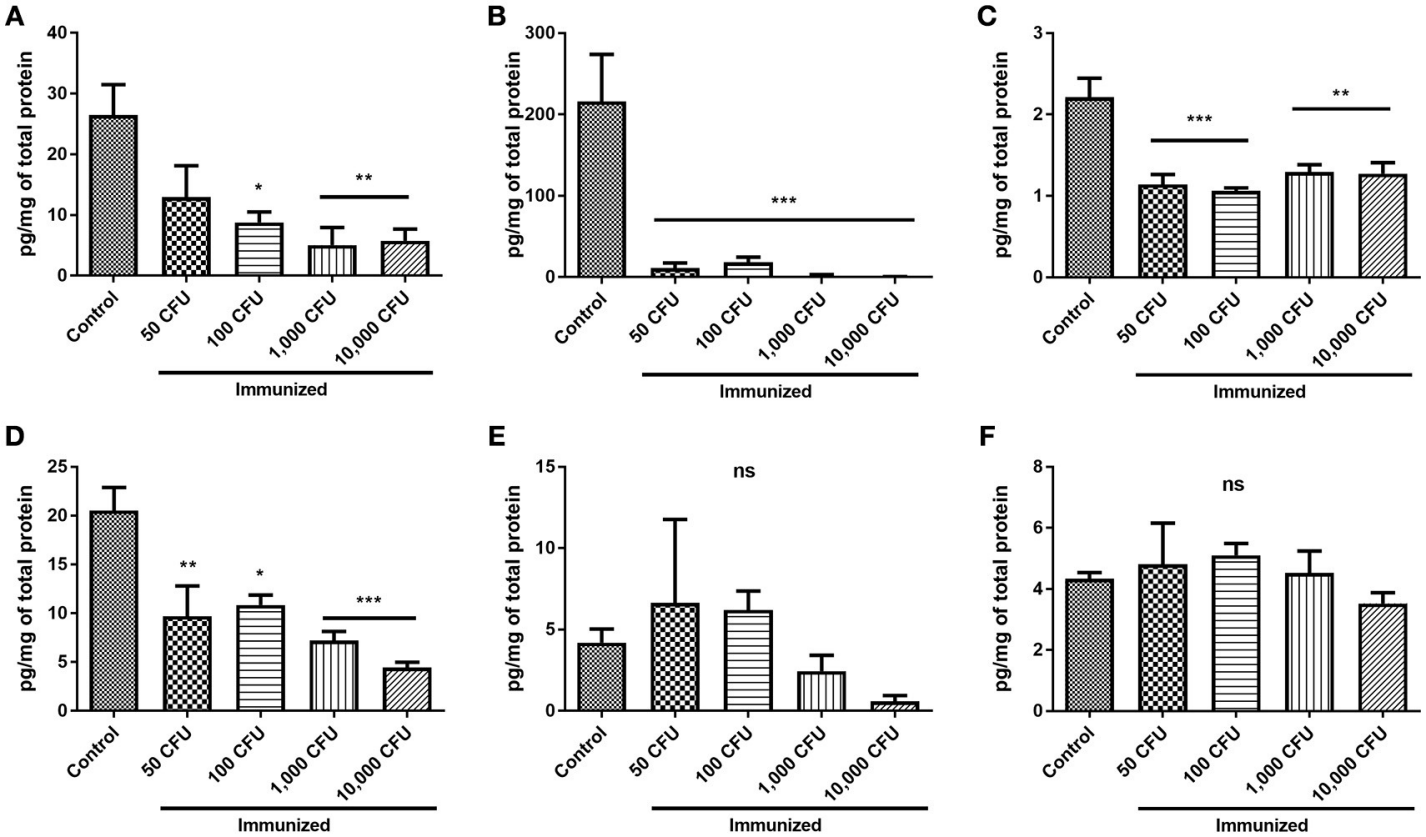
**Levels of splenic cytokines in control and immunized mice after challenge**. Spleens were harvested after euthanasia and lysed as described in Section Materials and Methods to analyze the levels of splenic cytokines/g of tissue. Cytokine levels were determined by Bio-Plex cytokine assay and reported as μg/mg of the total protein, determined by BCA. Levels of TNF **(A)**, INF-γ **(B)**, IL-4 **(C)**, and IL-10 **(D)**/g of tissue were significantly lower in mice immunized with *F. novicida* Δ1212–1218 than control mice. Levels of IL-12 (p70) **(E)** and IL-2 **(F)** were not significantly different between groups. ^*^p < 0.05, ^**^p < 0.01, ^***^p < 0.001.

Spleen lysate cytokine levels were determined as pg of cytokine/mg of total protein ± the standard error of the mean. Levels of TNF-α, INF-γ, IL-4, and IL-10 were significantly higher in the spleens of challenged control mice than in the spleens of challenged immunized mice (Figures 10A–D; *p*-values for cytokine levels of the group immunized with 10,000 CFU compared to the control mice were *p* < 0.01, < 0.001, < 0.01, and < 0.001 respectively*)*. There was no significant difference in the levels of IL-12(p70) and IL-2 produced by the spleens of control and immunized mice (Figures 10E,F; *p* ≥ 0.325), although there was detectably less IL-12(p70) made by mice immunized with 10,000 CFU of the mutant than controls (Figure 10E). GM-CSF levels were only detected in control mice and not in immunized mice, and IL-5 was not detected in any group. Overall, control mice exhibited hypercytokinemia compared to immunized mice.

## Discussion

*F. novicida* is reported to be non-encapsulated, whereas *F. tularensis* subspecies *tularensis* and *holarctica* are described as encapsulated (Sjöstedt, 2005; Elkins et al., 2007; Barker et al., 2009). The presence of a capsule around *F. tularensis* subspecies *tularensis* and *holarctica* is based on the observation of an electron dense material surrounding the cells by electron microscopy (Sandström et al., 1988; Cherwonogrodzky et al., 1994). Nevertheless, such “encapsulation” is only observed following specific growth conditions. Cherwonogrodzky et al. (1994) reported that subculture of LVS in defined broth medium, such as CDMB, followed by culture for several days on defined medium agar (CDMA), increased the amount of electron dense material surrounding the bacterial cells, and enhanced the virulence of these cells in mice. Previously, we used this serial passage method (adding to it, lowering the culture temperature to 32°C) to confirm that the electron dense material around LVS and type A strains is enhanced during such growth conditions, and that it consists predominately of protein, but also carbohydrate; it is now referred to as CLC (Bandara et al., 2011). *F. novicida* may have been described as non-encapsulated because it was not grown under the proper conditions to make a similar electron dense material visible. It has not been determined what component(s) of CDM enhance(s) expression of the CLC. However, spermine is present in the CDM and has been shown to induce extensive changes in gene expression, enabling *F. tularensis* to recognize its eukaryotic host environment (Carlson et al., 2009). Furthermore, mutagenesis of the gene putatively responsible for the spermine response attenuated both the LVS and Type A strain SchuS4 *in vivo* (Russo et al., 2011). Lowering the temperature to 32°C may also simulate environmental conditions more closely related to other hosts, such as ticks. Therefore, CDM may simulate signaling by the host environment for *F. tularensis* more closely than other media.

Passage of *F. novicida* in CDMB, followed by growth at 32°C on CDMA, enhanced surface expression of an electron dense material, which was not present around *F. novicida* cells grown to mid-log phase in shaking broth. This electron dense material appeared similar to the material surrounding both LVS (Bandara et al., 2011) and *F. tularensis* subspecies *tularensis*. The *F. novicida* extracellular material was composed of an array of proteins and a relatively small portion of carbohydrate (consisting of glucose, galactose, and mannose), similar to the CLC of LVS (Bandara et al., 2011). The electrophoretic protein profile of the *F. novicida*_P10 extract was also similar to the profile of the CLC from LVS and contained components of similar high molecular size. Differential staining further supported that this large molecular size material was glycosylated. Therefore, *F. novicida* did produce a CLC similar to the CLC of the more virulent subspecies.

A genetic locus with homology to the CLC glycosylation locus of LVS and Type A strains was also identified in *F. novicida* (Bandara et al., 2011; Thomas et al., 2011). Deletion of two glycosyltransferases (FTL_1423 and FTL_1422; a mannosyltransferase and galactosyltransferase, respectively) eliminated expression of the CLC in LVS. However, deletion of only one of the genes in this locus did not significantly affect glycosylation (Bandara et al., 2011). We have previously reported that deletion of only one glycosyltransferase gene from the capsule locus of *Actinobacillus pleuropneumoniae* did not completely eliminate capsule expression (Bandara et al., 2003). Furthermore, there was no significant difference in the carbohydrate content of the CLC from seven *F. novicida* TN mutants with one gene inactivated within the glycosylation locus and the parent strain. Therefore, the two genes homologous to those deleted in LVS were targeted for deletion (FTN_1212 and FTN_1213) using the same vector used for mutation of LVS. To our surprise, the allelic exchange process in *F. novicida* also resulted in loss of upstream genes FTN_1214–1218, but not downstream gene FTN_1211.

It is not clear why so many genes were affected by this allelic exchange procedure, but substantial identity (51%) between base pairs at FTN_1219 (the upstream region deleted in *F. novicida*) and FTL_1424 (the upstream gene in the LVS mutagenesis vector) was identified that may have accounted for the recombination event involving a vector with highly similar DNA of another subspecies. Nonetheless, all of the affected genes were within the glycosylation locus, and deletion of the additional genes should only affect glycosylation of proteins and the CLC.

The content of carbohydrate in crude CLC extracts of the subcultured mutant was significantly reduced compared to the subcultured parent, even though the LPS content was not affected in the mutant. However, the mutations did not significantly affect CLC protein content. Nonetheless, the mutation reduced both the amount of CLC observed, and the association of the CLC proteins with the *F. novicida* cell surface. When observed by electron microscopy, what little CLC was present around *F. novicida* Δ1212–1218_P10 was less adherent, scattered, and away from the bacterial cells, whereas the CLC of wild-type *F. novicida*_P10 covered the cells and was more directly associated with the bacterial surface. In *Haemophilus influenzae*, the adhesin HMW1 is glycosylated and this glycosylation is necessary to tether the protein to the bacterial surface: removal of the glycan disassociates the protein from the bacterium (Grass et al., 2003). Therefore, the carbohydrate component of the CLC may also contribute to close association of the CLC proteins to the bacterial surface and contribute to the aggregation of these proteins, as observed by electron microscopy. Enhanced expression of CLC may also promote binding of the bacteria to phagocytic cells and promote bacterial uptake.

Protein glycosylation appears to be widespread in *Francisella* species. Identical glycans have been identified that modify proteins that include PilA (Egge-Jacobsen et al., 2011), DsbA (Thomas et al., 2011; Balonova et al., 2012), and FTH_0069 (Balonova et al., 2012) in *F. tularensis* subspecies *holarctica* and *tularensis*. The glycan modifying these proteins is a hexasaccharide and the synthesis of this glycan has been linked to genes present in the putative CLC glycosylation locus of subspecies *tularensis* (Thomas et al., 2011). A multi-method approach of lectin enrichment, lectin blotting, and glycoprotein-specific staining was also used to identify up to 15 putative glycoproteins in *F. tularensis* subspecies *holarctica* extracts (Balonova et al., 2010). All of these 15 putative glycoproteins have homologs in *F. novicida* and some of the proteins are putative outer membrane proteins such as FopA and TUL4. Nevertheless, other than PilA, DsbA, and FTH_0069, no other *Francisella* proteins have been definitively confirmed as glycoproteins.

The differences noted by electron microscopy and virulence between LVS and *F. novicida* mutants lacking the same glycosyltransferases are minimal, but may be related to other mutations in LVS. There are multiple mutations in LVS that may contribute to the bacterium's attenuation in immunocompetent individuals, including multiple hypothetical proteins, outer membrane proteins, metabolism proteins, and more (Rohmer et al., 2006). LVS also contains a truncated version of PilA, which has been shown to be necessary for full virulence in both subspecies *holarctica* (Forslund et al., 2006) and *tularensis* (Forslund et al., 2010). In contrast, strain U112 is a wildtype strain of *F. tularensis* and is as virulent for mice as *F. tularensis* type A. In contrast, mice challenged IN with up to 1,000 times the LD_50_ of *F. novicida* Δ1212–1218 only developed subclinical infections. During a time course comparing an *F. novicida* Δ1212–1218 infection with that of an equal dose of the parent, *F. novicida* Δ1212–1218 did not proliferate in the tissues to the same extent as the wild-type strain, and was below detection level in tissues until day 3 post-challenge, whereas bacterial numbers of *F. novicida* U112 were one or more logs greater. *F. novicida* Δ1212–1218 was also unable to disseminate throughout the tissues as effectively as the parent. Therefore, protein glycosylation of the CLC played an important role in the ability of *F. novicida* to disseminate in the mouse, as previously described for LVS (Bandara et al., 2011). The observation that enhanced expression of CLC increases the virulence of *F. tularensis* (Cherwonogrodzky et al., 1994) and our current and previous findings that inability to glycosylate and fully produce CLC attenuates the bacteria (Bandara et al., 2011), indicates that the CLC contributes to *F. tularensis* virulence. The mechanism responsible for enhancing virulence is unknown, but may be related to resistance to innate host defenses, promoting macrophage uptake, or promoting escape from the phagosome to the cytosol (Golovliov et al., 2003).

It was not practical to attempt complementation of seven genes in *F. novicida* Δ1212–1218. Nonetheless, the mutant was transformed with shuttle vector pFNLTP6 (Bandara et al., 2011) containing the genes targeted for deletion. In data not shown this partially complemented strain contained more carbohydrate than the mutant, less than the parent, but was not significantly different from either. Mice inoculated with 1,000 CFU or more of the mutant expressing FTN_1212 and FTN_1213 demonstrated clinical symptoms that included ruffled fur, reduced activity, and weight loss, but the mice survived for 14 days, unlike mice challenged with the parent. Mice inoculated with 10,000 CFU of this partially complemented mutant had significantly more weight loss than mice inoculated with 10,000 CFU of *F. novicida* Δ1212–1218 (*p* < 0.005; data not shown). Thus, although as expected complementation of *F. novicida* Δ1212–1218 with only genes FTN_1212 and FTN_1213 did not restore full virulence to the mutant, these results showed that full glycosylation in *F. novicida* required more than one gene, and partial glycosylation and virulence could be restored despite the lack of several genes. Synthesis of a carbohydrate polymer requires many genes, including transferases, polymerase, phosphorylases, and proteins for export and transport of the polymer across membranes (Whitfield, 2006). BLAST analysis indicated that FTN_1212–1214 and FTN-1218 are putative glycosyltransferases. FTN_1216 encodes a hypothetical protein of yet unknown function. FTN-1215 may encode a protein responsible for capsule export and FTN_1217 for an ATP-binding membrane transporter; both are involved in polysaccharide export. Transport and synthesis of other polysaccharides in *F. tularensis*, such as LPS and O-antigen capsule, likely also utilize membrane transporters and transferases (the LPS core also contains mannose) that could substitute for some of the missing proteins, enabling partial restoration of CLC glycosylation.

Immunization with *F. novicida* Δ1212–1218 provided partial protection to mice against IN challenge with the parent strain. This incomplete protection may be due to the inability of *F. novicida* Δ1212–1218 to effectively disseminate throughout the host and persist long enough to induce a more protective immune response, or the missing carbohydrate may be an important antigen for protective immunity. *F. tularensis* and *F. novicida* are facultative intracellular pathogens, and a robust cellular immune response is necessary to effectively clear the infection (Baron et al., 2007; Pechous et al., 2009; Shen et al., 2010). Immunodominant antigens such as the O-antigen provide partial protection against aerosolized challenge with virulent Type B strains, but not Type A strains (Conlan et al., 2002). Immunization of mice with recombinant FopA enclosed in liposomes can afford partial protection against challenge with LVS, but not against SchuS4 (Hickey et al., 2011). These studies suggest that humoral immunity may play an ancillary role in protection against *F. tularensis*. The disruption of the CLC glycan may result in instability of surface antigens surrounding the bacterium resulting in the loss of important antigenic epitopes necessary to stimulate supportive humoral immunity.

In mice, outward clinical signs of tularemia are delayed 2–3 days following infection with *Francisella*, but the disease quickly progresses and becomes lethal (Elkins et al., 2007; Mares et al., 2008; Cowley and Elkins, 2011). The pathology of tularemia is largely due to tissue damage following severe inflammation and hypercytokinemia that occurs after *Francisella* has replicated extensively (Mares et al., 2008; Sharma et al., 2009a,b, 2011). In this study, control mice infected with *F. novicida* did not show clinical signs until ~48 h after infection and became moribund between 72 and 96 h post-infection. Disease in the challenged, immunized mice did not advance as rapidly as in the control mice, and the infection in mice challenged with *F. novicida* Δ1212–1218 was subclinical. Sharma et al. (2009a,b) reported that mice challenged with *F. novicida* IN become severely septic and hypercytokinemic, whereas mice challenged with a *F. novicida* mutant lacking a 58-kDa protein produce lower levels of pro-inflammatory cytokines and do not succumb to disease. Mice immunized with *F. novicida* Δ1212–1218 and then challenged with the parent also exhibit reduced levels of pro-inflammatory cytokines, such as TNF-α, compared to control mice (Sharma et al., 2009a). The level of tissue destruction in the lungs correlates with the levels of pro-inflammatory cytokines; the hypercytokinemic control mice exhibit more severe tissue destruction than the immunized mice after challenge (Sharma et al., 2009a). Our results were similar to those of Sharma et al. (2009a) in regard to differences in cytokine responses between immunized and control mice. Immunized mice from both studies produced significantly less TNF-α, IL-10, and GM-CSF after challenge than control mice after challenge. The spike in TNF-α that precedes morbidity may be more detrimental to the host and contribute to sepsis instead of effectively controlling the bacterial infection (Mares et al., 2008; Sharma et al., 2009a,b, 2011). Bakshi et al. showed that immunization with an attenuated LVS stain, *sodB*_*FT*_, results in a more controlled release of pro-inflammatory cytokines after challenge than unimmunized control mice (Bakshi et al., 2008). Higher production of pro-inflammatory cytokines in mice correlates with greater amounts of inflammation and tissue destruction; a delayed response in cytokine production followed by massive up-regulation is a sign of severe tularemia (Metzger et al., 2007; Bakshi et al., 2008). In our study, unlike control mice, mice inoculated with the mutant were not hypercytokinemic after challenge and the spleens of these mice did not exhibit the same level of inflammation and tissue destruction as the spleens of mice inoculated with the parent. Although, immunization of mice with the mutant was not fully protective, the partial protection afforded may be attributed to a more regulated immune response in immunized mice than in control mice after challenge. Interferon gamma (IFN-γ) has been implicated as essential for controlling the replication of *F. tularensis* during the initial infection. Both Sharma et al. (2009a,b) and Bakshi et al. (2006) reported that immunized mice surviving a lethal challenge exhibit a significant increase in IFN-γ compared to controls after infection, followed by a return to baseline levels. In this study, the spleens of immunized mice did not contain significantly higher levels of IFN-γ than control mice. The lack of enhanced expression of IFN-γ in mice immunized with *F. novicida* Δ1212–1218 may explain the lack of full protection following challenge with the parent. The LD_50_ of *F. novicida* for mice by the respiratory route is <10 CFUs (Kieffer et al., 2003; Lauriano et al., 2004; Cong et al., 2009). Therefore, it was not possible to compare the immune response of mice immunized with the mutant to that of a sublethal dose of the parent.

In summary, we have shown that, as for *F. tularensis, F. novicida* produced a CLC when grown under conditions that enhanced expression of the electron dense CLC in LVS. The mixture of glycosylated proteins in the *F. tularensis* CLC have been partially identified (manuscript in preparation). Furthermore, the use of *F. novicida* and the immense transposon library available may further aid in identifying these proteins and the role of CLC in pathogenesis. Loss of surface protein glycosylation and the deficiency of surface-associated CLC on the bacteria attenuated *F. novicida* and LVS in a mouse model, and such mutants conferred partial protective immunity against challenge with virulent strains. Mutagenesis of these glycosyltransferase genes in a Type A strain of *F. tularensis* is in progress to determine if such a mutant would also be attenuated and induce protective immunity against type A challenge.

## Ethics statement

This study was carried out in accordance with the recommendations of the U.S. Government Principles for the Utilization and Care of Vertebrate Animals Used in Testing, Research and Training, The Animal Welfare Act, The Public Health Service Policy on Humane Care and Use of Laboratory Animals, and Virginia Tech Policies Governing the Use of Animals in Research and Teaching, by the Virginia Tech Institutional Animal Care and Use Committee. The protocol was approved by the Virginia Tech Institutional Animal Care and Use Committee; Assurance number A-3208-01, expiration date 7/31/2017.

## Author contributions

KF conceived and performed most of the experiments, wrote the initial draft of the manuscript, contributed to revisions, and read/edited the final draft. AC performed some experiments, wrote part of the manuscript, and read/edited the final draft. NM contributed to generating the allelic exchange mutant, wrote part of the manuscript, and read/edited the final draft. TC performed the clinical pathology and read/edited the final draft. TI conceived the experiments, revised the manuscript, and carried out editing of the final draft.

### Conflict of interest statement

The authors declare that the research was conducted in the absence of any commercial or financial relationships that could be construed as a potential conflict of interest.
