# Corrigendum: Glycosylation of a Capsule-Like Complex (CLC) by *Francisella novicida* Is Required for Virulence and Partial Protective Immunity in Mice

**DOI:** 10.3389/fmicb.2019.01090

**Published:** 2019-05-15

**Authors:** Kelly C. Freudenberger Catanzaro, Anna E. Champion, Nrusingh Mohapatra, Thomas Cecere, Thomas J. Inzana

**Affiliations:** ^1^Department of Biomedical Sciences and Pathobiology, Center for Molecular Medicine and Infectious Diseases, Virginia-Maryland College of Veterinary Medicine, Virginia Tech, Blacksburg, VA, United States; ^2^Department of Biomedical Sciences, Virginia Tech Carilion School of Medicine, Roanoke, VA, United States

**Keywords:** *Francisella novicida*, capsule-like complex, virulence in mice, tularemia, glycosylation, protective immunity

In the original article, there was a mistake in the legend for Figure 6 as published. Figure 6 has 5 lanes, but only 4 are described in the legend. The lane that is not described is lane 3, which should list “3, *F. novicida* Δ1212–1218[1212-1213+].” As stated the description of lanes 3 and 4 are also wrong, as they should be listed as lanes 4 and 5. The correct legend appears below. The authors apologize for this error and state that this does not change the scientific conclusions of the article in any way. The original article has been updated.

“Figure 6 | Carbohydrate and protein content of urea extracts from *F. novicida* and LVS strains. All strains were subcultured in CDMB and grown on CDMA at 32°C to enhance putative CLC production. The surface material was extracted using 1 M urea, and the crude CLC extracts were analyzed for carbohydrate content by the anthrone assay and for protein content by the BCA assay. The carbohydrate content of the extract from *F. novicida* Δ11212–1218_P10 contained significantly less carbohydrate than that of *F. novicida*_P10 (*p* = 0.02). The protein content between subcultured strains of *F. novicida* or between strains of LVS was not statistically different. Lanes: 1, *F. novicida*_P10; 2, *F. novicida* Δ11212–1218_P10; 3, *F. novicida* Δ1212–1218[1212–1213+]; 4, LVS_P10; 5, LVSΔ1422-1423_P10. Different letters above the bars indicate significant differences between the means when one-way ANOVA and Tukey's *post-hoc* were performed (*p* < 0.05). Identical uppercase letters indicate that there is no significant difference between the means. The value of bar marked with “A” is significantly different from the values of bars marked with “B” or “C.” The values of bars marked “AB” are not statistically different from the values of bars marked with “A” or with “B,” but are statistically different from a bar marked with “C.””

