# Supplementary material for: Glycosylation of a Capsule-Like Complex (CLC) by Francisella novicida Is Required for Virulence and Partial Protective Immunity in Mice
Source: Front Microbiol. 2017 May 30;8:935. doi: 10.3389/fmicb.2017.00935 (PMC5447757; doi:10.3389/fmicb.2017.00935)
Supplement: Supplementary file 1 [file Table1.PDF]

Supplemental Tables and Figures

Table S1. Oligonucleotide primers used for RT-PCR.

Primer	Sequence (5’ → 3’)
FTN_1211_F	GAGTTTGGTTCAACTATGCTTGG
FTN_1212_F_BamHI	CGCGGATCCATGCCAAAATTATTAATAGATACGCGC
FTN_1213_F_Xbal	CTAGTCTAGAATCGATTGTTGTTTCAAGTTTTGATAATGATTA<br>AAAATAATAGGAGTTAAAAATGTACAATCTTAATTATAAGCA<br>GCTAATATCTATAATC
FTN_1214_F	GGCTCTACTGATAACTCTCTGG
FTN_1215_F	GTTCTAACTGGATGAGAGGGATG
FTN_1216_F	CCGGAGATGGTTTCAGGTAAA
FTN_1217_F	AAGAAGTACACCGACACTTATCC
FTN_1218_F	GCTATCGATTGCTACGGCTAAA
FTN_1219_F	GAGATTATCAAGTTGTGGTGGTAGA
FTN_1220_F	CTAAAGCTGAGGCTGCTAAGT
FTN_1221_F	CTTCTATACTCTCTGCCGATCTTG
FTN_1211_R	CTCCTAGCTAAATACTTACCCTCAAA

**FTN_1212_R_XmaI**	**TCCCCCCGGGCTATAATAAATTAAGCTTTTTAAGCTCATCTT<br>GGAC**
**FTN_1213_R_XmaI**	**TCCCCCCGGGTTAACTTCTAGTAATTCTTTTTTGTTTGAGTGAG**
**FTN_1214_R**	**CTACTTAATAACCCTGCTGAATCAA**
**FTN_1215_R**	**CTTCAAAGCCTAACTGTGATGTC**
**FTN_1216_R**	**CCAATCTCTATCTTAGGCTTCCAT**
**FTN_1217_R**	**GTGCCGTATAGAAGCCCATTA**
**FTN_1218_R**	**TGATCGCCATCTCCAACTATAAC**
**FTN_1219_R**	**CTCTCACTCCCGTACCATCTAT**
**FTN_1220_R**	**ACTTGCGCGTAACCAGTAATA**
**FTN_1221_R**	**GGATTTACGCCACCATCTATCT**

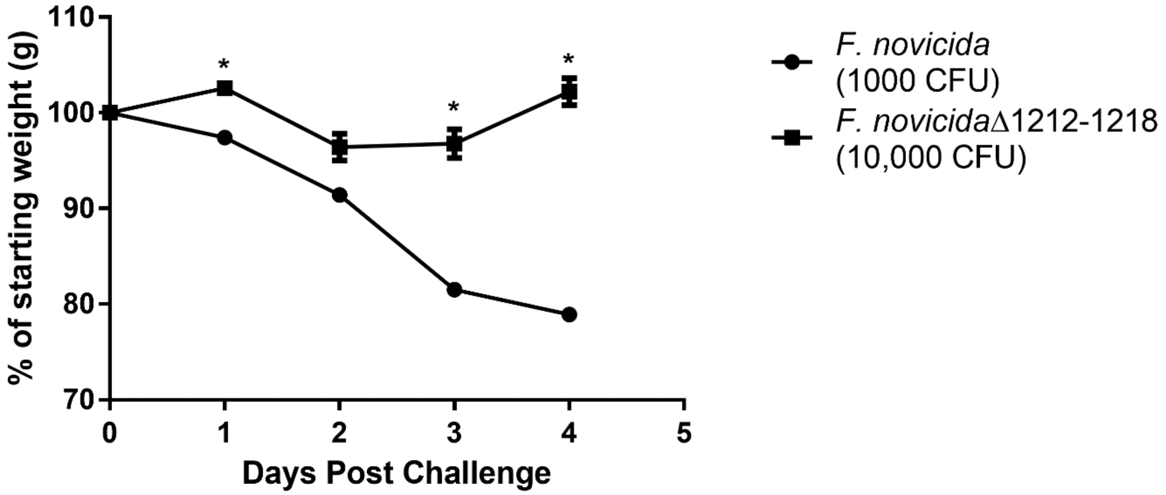

Fig. S1. Weight loss of mice challenged with *F. novicida* or *F. novicida*Δ1212-1218. The percentage of weight loss based on starting weight was determined for each group of mice challenged with *F. novicida* or *F. novicida*Δ1212-1218. Multiple t-tests using the Holm-Sidak method for correction were used to determine statistical differences between groups at specific time points. Mice inoculated with *F. novicida* (●) were euthanized by four days post challenge. Mice inoculated with *F. novicida*Δ1212-1218 (■) had significantly less weight loss at 1, 3, and 4 days post challenge ($p < 0.005$, *).
