# Supplementary material for: Glycosylation of a Capsule-Like Complex (CLC) by Francisella novicida Is Required for Virulence and Partial Protective Immunity in Mice
Source: Front Microbiol. 2017 May 30;8:935. doi: 10.3389/fmicb.2017.00935 (PMC5447757; doi:10.3389/fmicb.2017.00935)
Supplement: Supplementary file 2 [file Table2.pdf]

Table S2. Carbohydrate Content of *F. novicida* Transposon Mutants Compared to Parent

**Strain**	**Transposon Insertion Location**	**Carbohydrate of Crude Urea Extract (µg/gram of bacterial wet weight) (n = 3)[a]**
*F. novicida*_P10[b]	N/A	4078 ± 415
tnfn1_pw060323p05q162	FTN_1212	3329 ± 596
tnfn1_pw060323p03q152	FTN_1213	2866 ± 613
tnfn1_pw060328p06q149	FTN_1214	3831 ± 544
tnfn1_pw060323p05q110	FTN_1215	3532 ± 545
tnfn1_pw060420p04q184	FTN_1216	3392 ± 641
tnfn1_pw060418p03q107	FTN_1218	3171 ± 393
tnfn1_pw060323p07q127	FTN_1219	3597 ± 192

[a] Comparison of carbohydrate content of urea extracts of *F. novicida*_P10 and subcultured *F. novicida* transposon mutants was done with a One-Way ANOVA. No significant differences were found (p-value > 0.05).

[b]The parent and all mutants were subcultured daily in CDMB for 10 days, followed by growth on CDMA at 32°C to enhance CLC expression.
